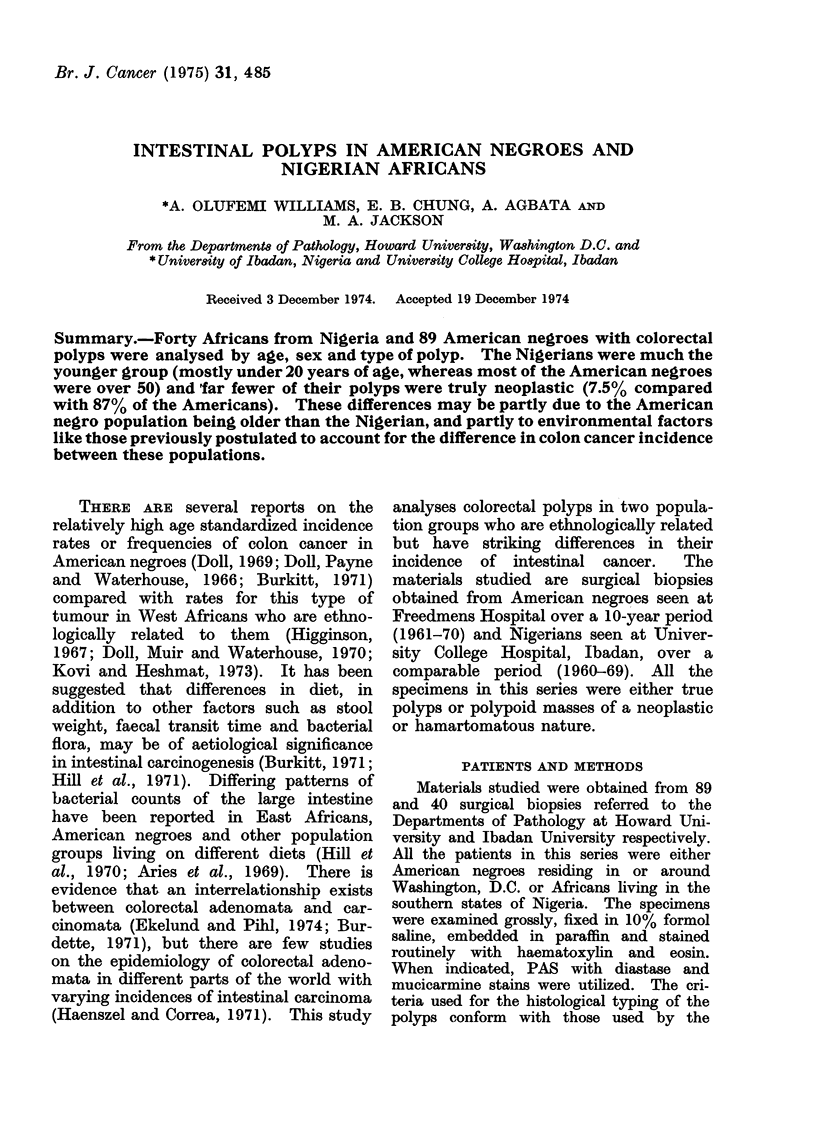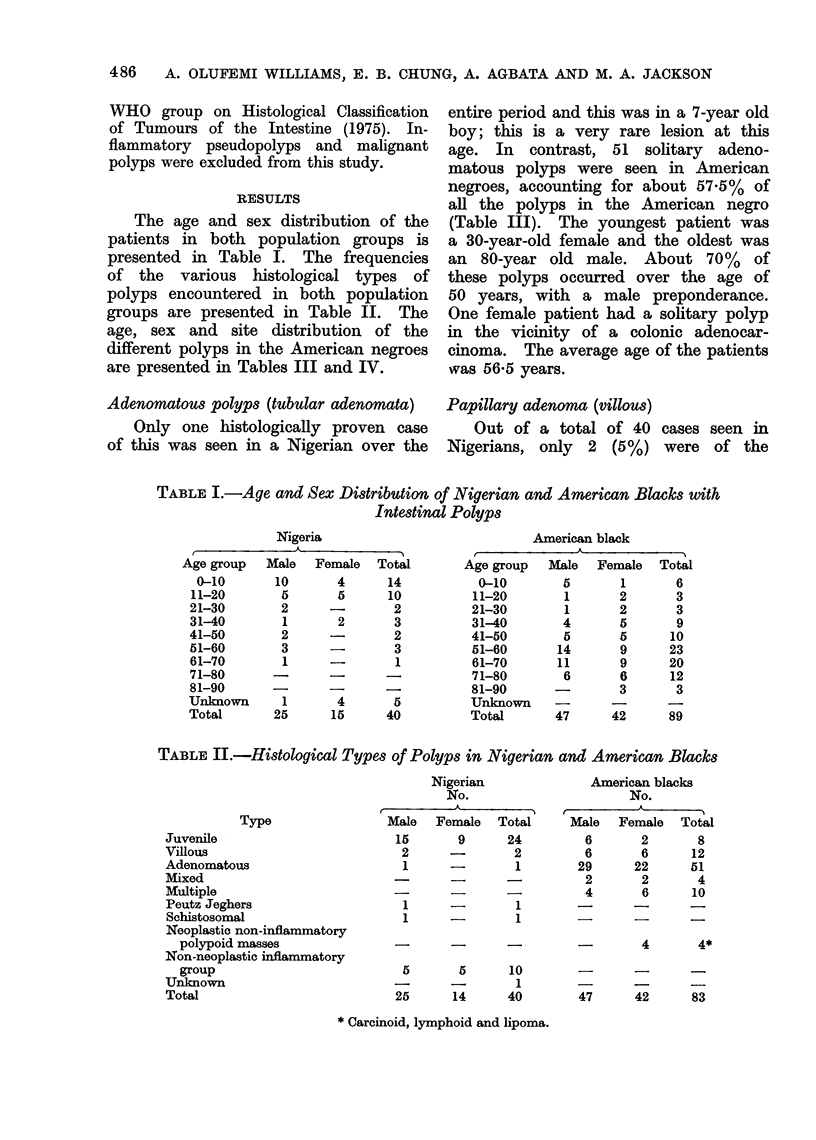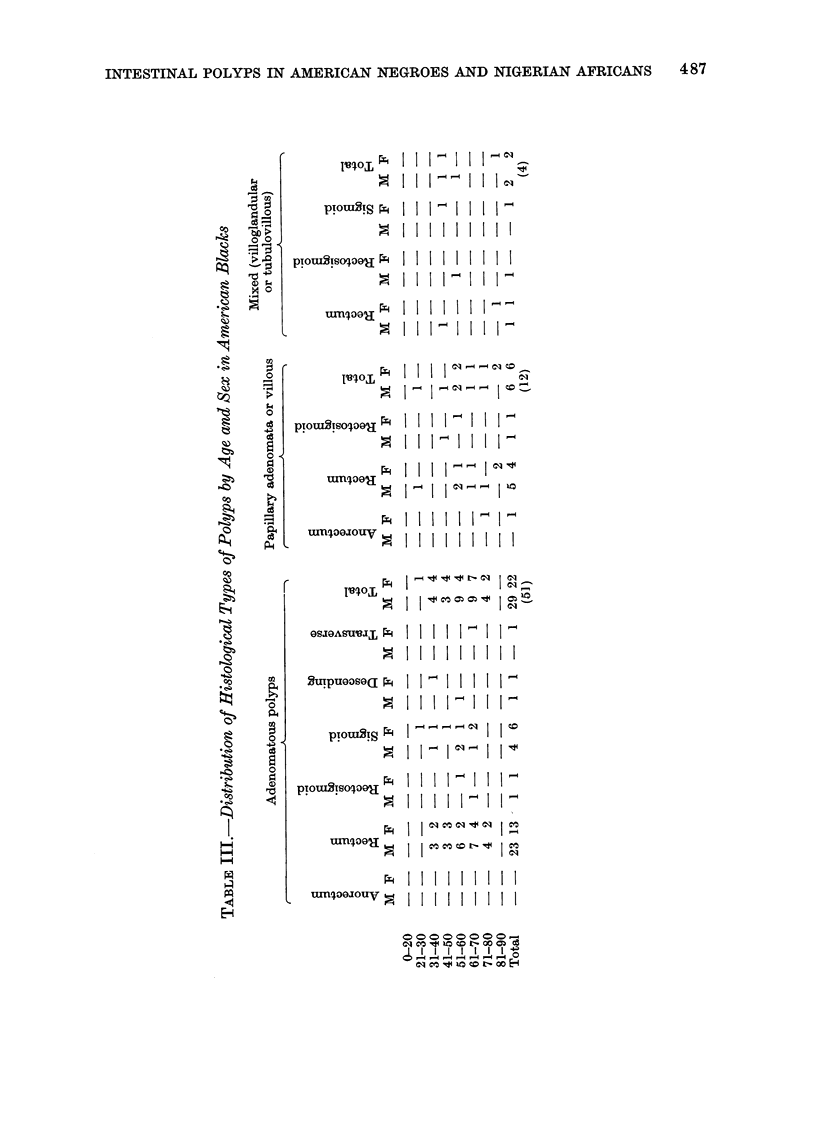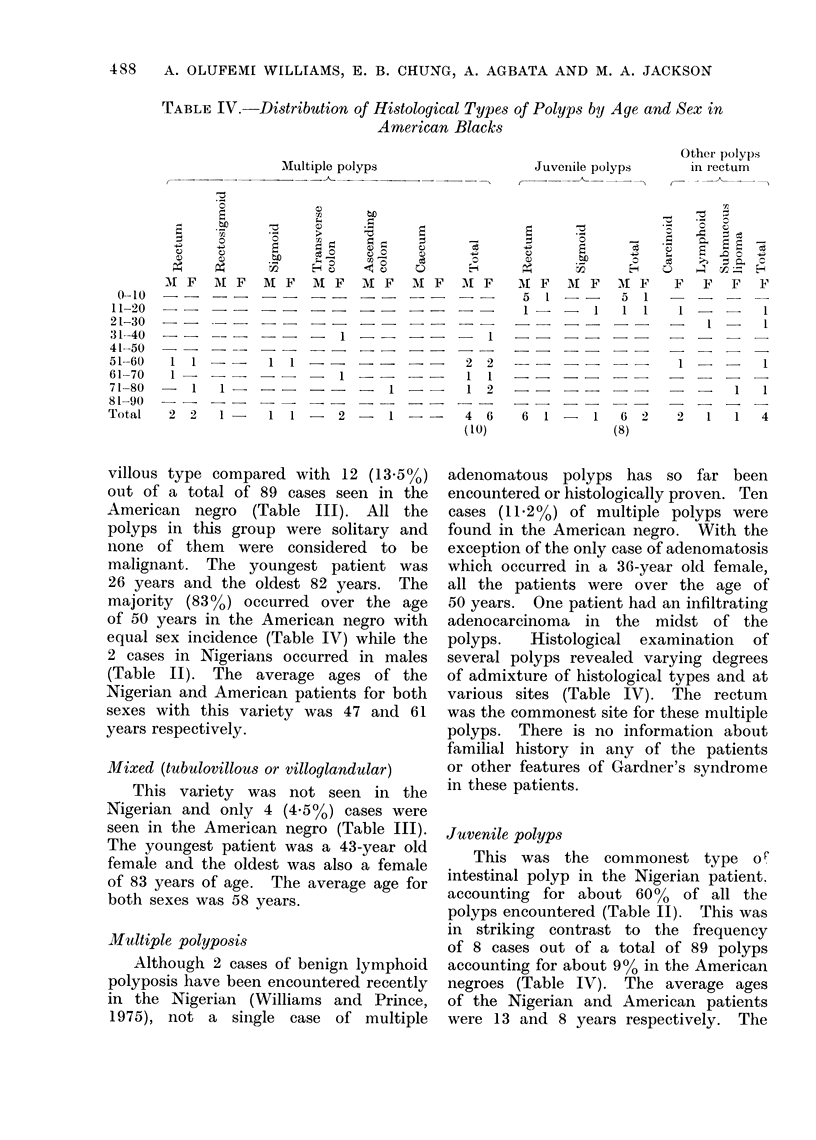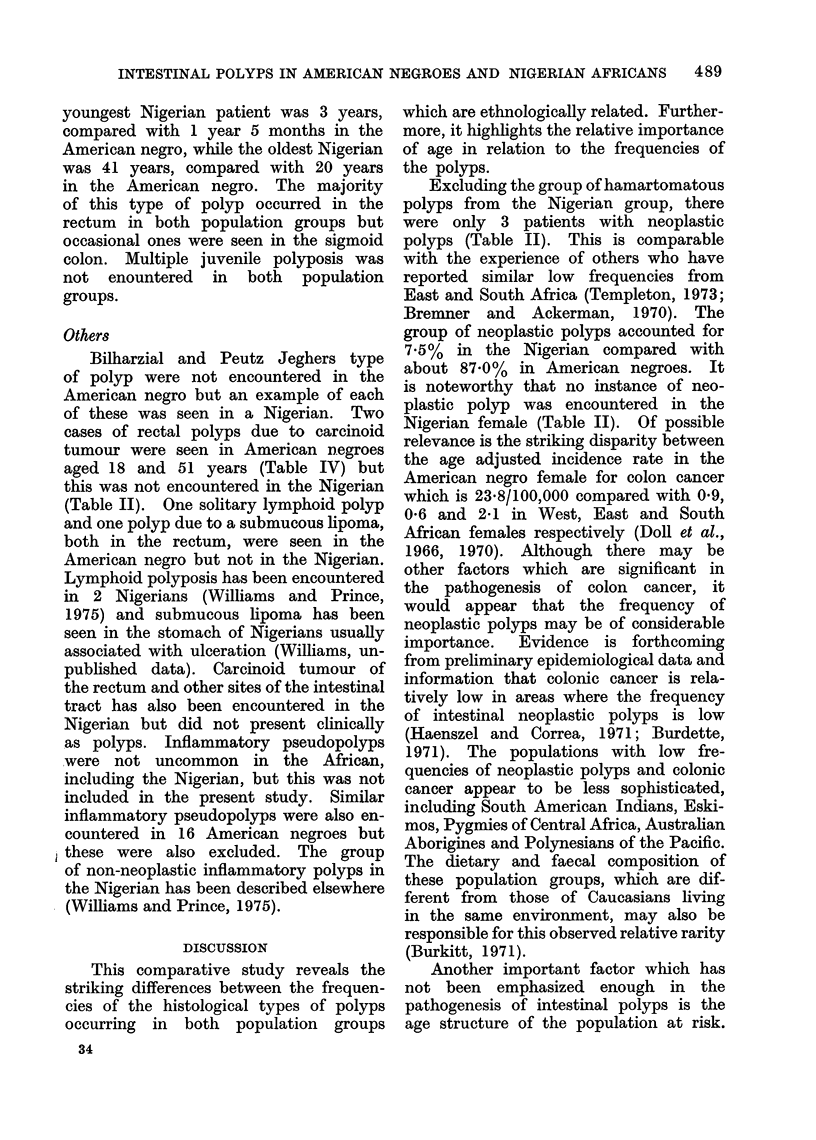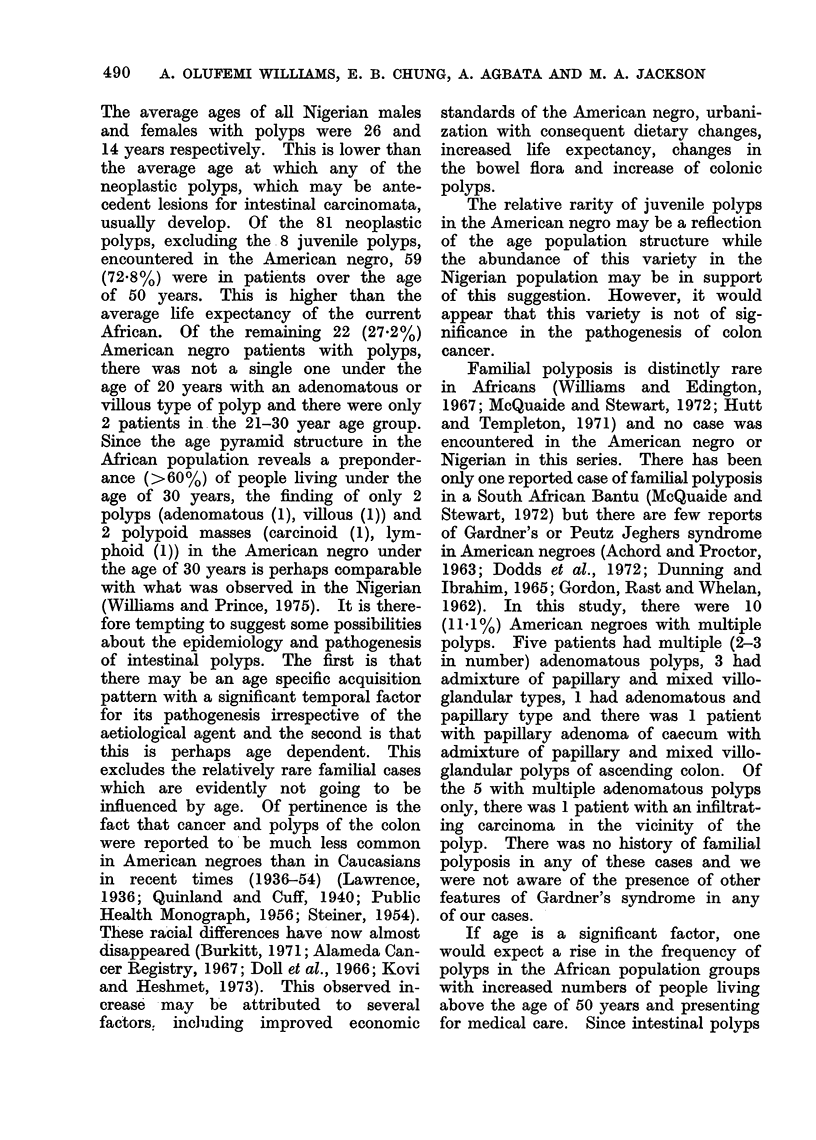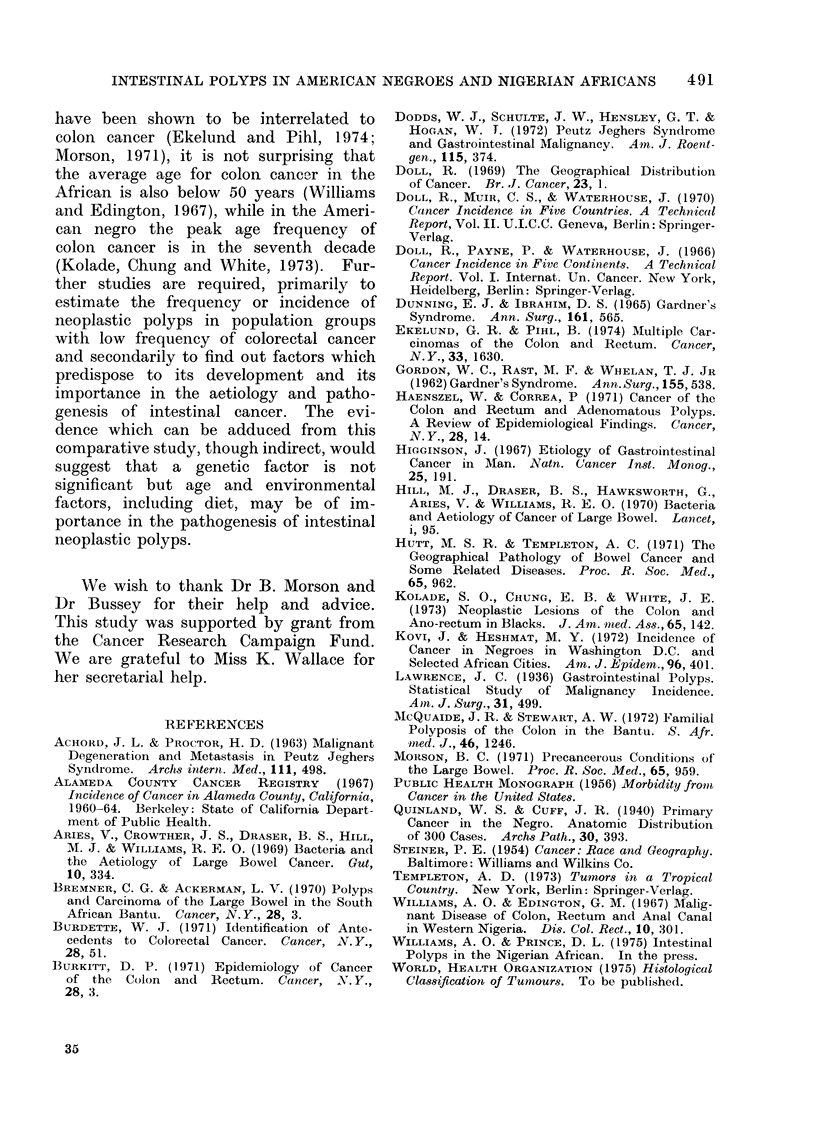# Intestinal polyps in American negroes and Nigerian africans.

**DOI:** 10.1038/bjc.1975.88

**Published:** 1975-04

**Authors:** A. O. Williams, E. B. Chung, A. Agbata, M. A. Jackson

## Abstract

Forty Africans from Nigeria and 89 American negroes with colorectal polyps were analysed by age, sex and type of polyp. The Nigerians were much the younger group (mostly under 20 years of age, whereas most of the American negroes were over 50) and far fewer of their polyps were truly neoplastic (7.5% compared with 87% of the Americans). These differences may be partly due to the American negro population being older than the Nigerian, and partly to environmental factors like those previously postulated to account for the difference in colon cancer incidence between these populations.


*Br. J. Cancer* (1975) **31**, 485

### INTESTINAL POLYPS IN AMERICAN NEGROES AND NIGERIAN AFRICANS

*A. OLUFEMI WILLIAMS, E. B. CHUNG, A. AGBATA AND M. A. JACKSON

*From the Departments of Pathology, Howard University, Washington D.C. and *University of Ibadan, Nigeria and University College Hospital, Ibadan*

Received 3 December 1974. Accepted 19 December 1974

**Summary.—Forty Africans from Nigeria and 89 American negroes with colorectal polyps were analysed by age, sex and type of polyp. The Nigerians were much the younger group (mostly under 20 years of age, whereas most of the American negroes were over 50) and far fewer of their polyps were truly neoplastic (7.5% compared with 87% of the Americans). These differences may be partly due to the American negro population being older than the Nigerian, and partly to environmental factors like those previously postulated to account for the difference in colon cancer incidence between these populations.**

THERE ARE several reports on the relatively high age standardized incidence rates or frequencies of colon cancer in American negroes (Doll, 1969; Doll, Payne and Waterhouse, 1966; Burkitt, 1971) compared with rates for this type of tumour in West Africans who are ethnologically related to them (Higginson, 1967; Doll, Muir and Waterhouse, 1970; Kovi and Heshmat, 1973). It has been suggested that differences in diet, in addition to other factors such as stool weight, faecal transit time and bacterial flora, may be of aetiological significance in intestinal carcinogenesis (Burkitt, 1971; Hill *et al.*, 1971). Differing patterns of bacterial counts of the large intestine have been reported in East Africans, American negroes and other population groups living on different diets (Hill *et al.*, 1970; Aries *et al.*, 1969). There is evidence that an interrelationship exists between colorectal adenomata and carcinomata (Ekelund and Pihl, 1974; Burdette, 1971), but there are few studies on the epidemiology of colorectal adenomata in different parts of the world with varying incidences of intestinal carcinoma (Haenszel and Correa, 1971). This study analyses colorectal polyps in two population groups who are ethnologically related but have striking differences in their incidence of intestinal cancer. The materials studied are surgical biopsies obtained from American negroes seen at Freedmens Hospital over a 10-year period (1961–70) and Nigerians seen at University College Hospital, Ibadan, over a comparable period (1960–69). All the specimens in this series were either true polyps or polypoid masses of a neoplastic or hamartomatous nature.

### PATIENTS AND METHODS

Materials studied were obtained from 89 and 40 surgical biopsies referred to the Departments of Pathology at Howard University and Ibadan University respectively. All the patients in this series were either American negroes residing in or around Washington, D.C. or Africans living in the southern states of Nigeria. The specimens were examined grossly, fixed in 10% formol saline, embedded in paraffin and stained routinely with haematoxylin and eosin. When indicated, PAS with diastase and mucicarmine stains were utilized. The criteria used for the histological typing of the polyps conform with those used by the

WHO group on Histological Classification of Tumours of the Intestine (1975). Inflammatory pseudopolyps and malignant polyps were excluded from this study.

### RESULTS

The age and sex distribution of the patients in both population groups is presented in Table I. The frequencies of the various histological types of polyps encountered in both population groups are presented in Table II. The age, sex and site distribution of the different polyps in the American negroes are presented in Tables III and IV.

### *Adenomatous polyps (tubular adenomata)*

Only one histologically proven case of this was seen in a Nigerian over the entire period and this was in a 7-year old boy; this is a very rare lesion at this age. In contrast, 51 solitary adenomatous polyps were seen in American negroes, accounting for about 57·5% of all the polyps in the American negro (Table III). The youngest patient was a 30-year-old female and the oldest was an 80-year old male. About 70% of these polyps occurred over the age of 50 years, with a male preponderance. One female patient had a solitary polyp in the vicinity of a colonic adenocarcinoma. The average age of the patients was 56·5 years.

### *Papillary adenoma (villous)*

Out of a total of 40 cases seen in Nigerians, only 2 (5%) were of the

TABLE I.—*Age and Sex Distribution of Nigerian and American Blacks with Intestinal Polyps*

| Nigeria | | | | American black | | | |
|---|---|---|---|---|---|---|---|
| Age group | Male | Female | Total | Age group | Male | Female | Total |
| 0–10 | 10 | 4 | 14 | 0–10 | 5 | 1 | 6 |
| 11–20 | 5 | 5 | 10 | 11–20 | 1 | 2 | 3 |
| 21–30 | 2 | — | 2 | 21–30 | 1 | 2 | 3 |
| 31–40 | 1 | 2 | 3 | 31–40 | 4 | 5 | 9 |
| 41–50 | 2 | — | 2 | 41–50 | 5 | 5 | 10 |
| 51–60 | 3 | — | 3 | 51–60 | 14 | 9 | 23 |
| 61–70 | 1 | — | 1 | 61–70 | 11 | 9 | 20 |
| 71–80 | — | — | — | 71–80 | 6 | 6 | 12 |
| 81–90 | — | — | — | 81–90 | — | 3 | 3 |
| Unknown | 1 | 4 | 5 | Unknown | — | — | — |
| Total | 25 | 15 | 40 | Total | 47 | 42 | 89 |

TABLE II.—*Histological Types of Polyps in Nigerian and American Blacks*

| | Nigerian No. | | | American blacks No. | | |
|---|---|---|---|---|---|---|
| Type | Male | Female | Total | Male | Female | Total |
| Juvenile | 15 | 9 | 24 | 6 | 2 | 8 |
| Villous | 2 | — | 2 | 6 | 6 | 12 |
| Adenomatous | 1 | — | 1 | 29 | 22 | 51 |
| Mixed | — | — | — | 2 | 2 | 4 |
| Multiple | — | — | — | 4 | 6 | 10 |
| Peutz Jeghers | 1 | — | 1 | — | — | — |
| Schistosomal | 1 | — | 1 | — | — | — |
| Neoplastic non-inflammatory polypoid masses | — | — | — | — | 4 | 4* |
| Non-neoplastic inflammatory group | 5 | 5 | 10 | — | — | — |
| Unknown | — | — | 1 | — | — | — |
| Total | 25 | 14 | 40 | 47 | 42 | 83 |

* Carcinoid, lymphoid and lipoma.

TABLE III.—*Distribution of Histological Types of Polyps by Age and Sex in American Blacks*

| | Adenomatous polyps | | | | | | | | | | | | | | Papillary adenomata or villous | | | | | | | | Mixed (villoglandular or tubulovillous) | | | | | | | |
|---|---|---|---|---|---|---|---|---|---|---|---|---|---|---|---|---|---|---|---|---|---|---|---|---|---|---|---|---|---|---|
| | Anorectum | | Rectum | | Rectosigmoid | | Sigmoid | | Descending | | Transverse | | Total | | Anorectum | | Rectum | | Rectosigmoid | | Total | | Rectum | | Rectosigmoid | | Sigmoid | | Total | |
| | M | F | M | F | M | F | M | F | M | F | M | F | M | F | M | F | M | F | M | F | M | F | M | F | M | F | M | F | M | F |
| 0–20 | — | — | — | — | — | — | — | — | — | — | — | — | — | — | — | — | — | — | — | — | — | — | — | — | — | — | — | — | — | — |
| 21–30 | — | — | — | — | — | — | — | 1 | — | — | — | — | — | 1 | — | — | 1 | — | — | — | 1 | — | — | — | — | — | — | — | — | — |
| 31–40 | — | — | 3 | 2 | — | — | 1 | 1 | — | 1 | — | — | 4 | 4 | — | — | — | — | — | — | — | — | — | — | — | — | — | — | — | — |
| 41–50 | — | — | 3 | 3 | — | — | — | 1 | — | — | — | — | 3 | 4 | — | — | — | — | 1 | — | 1 | — | 1 | — | — | — | — | 1 | 1 | 1 |
| 51–60 | — | — | 6 | 2 | — | 1 | 2 | 1 | 1 | — | — | — | 9 | 4 | — | — | 2 | 1 | — | 1 | 2 | 2 | — | — | 1 | — | — | — | 1 | — |
| 61–70 | — | — | 7 | 4 | 1 | — | 1 | 2 | — | — | — | 1 | 9 | 7 | — | — | 1 | 1 | — | — | 1 | 1 | — | — | — | — | — | — | — | — |
| 71–80 | — | — | 4 | 2 | — | — | — | — | — | — | — | — | 4 | 2 | — | 1 | 1 | — | — | — | 1 | 1 | — | — | — | — | — | — | — | — |
| 81–90 | — | — | — | — | — | — | — | — | — | — | — | — | — | — | — | — | — | 2 | — | — | — | 2 | — | 1 | — | — | — | — | — | 1 |
| Total | — | — | 23 | 13 | 1 | 1 | 4 | 6 | 1 | 1 | — | 1 | 29 | 22 | — | 1 | 5 | 4 | 1 | 1 | 6 | 6 | 1 | 1 | 1 | — | — | 1 | 2 | 2 |
| | | | | | | | | | | | | | (51) | | | | | | | | (12) | | | | | | | | (4) | |

TABLE IV.—*Distribution of Histological Types of Polyps by Age and Sex in American Blacks*

| | Multiple polyps | | | | | | | | | | | | | | Juvenile polyps | | | | | | Other polyps in rectum | | | |
|---|---|---|---|---|---|---|---|---|---|---|---|---|---|---|---|---|---|---|---|---|---|---|---|---|
| | Rectum | | Rectosigmoid | | Sigmoid | | Transverse colon | | Ascending colon | | Caecum | | Total | | Rectum | | Sigmoid | | Total | | Carcinoid | Lymphoid | Submucous lipoma | Total |
| | M | F | M | F | M | F | M | F | M | F | M | F | M | F | M | F | M | F | M | F | F | F | F | F |
| 0–10 | — | — | — | — | — | — | — | — | — | — | — | — | — | — | 5 | 1 | — | — | 5 | 1 | — | — | — | — |
| 11–20 | — | — | — | — | — | — | — | — | — | — | — | — | — | — | 1 | — | — | 1 | 1 | 1 | 1 | — | — | 1 |
| 21–30 | — | — | — | — | — | — | — | — | — | — | — | — | — | — | — | — | — | — | — | — | — | 1 | — | 1 |
| 31–40 | — | — | — | — | — | — | — | 1 | — | — | — | — | — | 1 | — | — | — | — | — | — | — | — | — | — |
| 41–50 | — | — | — | — | — | — | — | — | — | — | — | — | — | — | — | — | — | — | — | — | — | — | — | — |
| 51–60 | 1 | 1 | — | — | 1 | 1 | — | — | — | — | — | — | 2 | 2 | — | — | — | — | — | — | 1 | — | — | 1 |
| 61–70 | 1 | — | — | — | — | — | — | 1 | — | — | — | — | 1 | 1 | — | — | — | — | — | — | — | — | — | — |
| 71–80 | — | 1 | 1 | — | — | — | — | — | — | 1 | — | — | 1 | 2 | — | — | — | — | — | — | — | — | 1 | 1 |
| 81–90 | — | — | — | — | — | — | — | — | — | — | — | — | — | — | — | — | — | — | — | — | — | — | — | — |
| Total | 2 | 2 | 1 | — | 1 | 1 | — | 2 | — | 1 | — | — | 4 | 6 | 6 | 1 | — | 1 | 6 | 2 | 2 | 1 | 1 | 4 |
| | | | | | | | | | | | | | (10) | | | | | | (8) | | | | | |

villous type compared with 12 (13·5%) out of a total of 89 cases seen in the American negro (Table III). All the polyps in this group were solitary and none of them were considered to be malignant. The youngest patient was 26 years and the oldest 82 years. The majority (83%) occurred over the age of 50 years in the American negro with equal sex incidence (Table IV) while the 2 cases in Nigerians occurred in males (Table II). The average ages of the Nigerian and American patients for both sexes with this variety was 47 and 61 years respectively.

*Mixed (tubulovillous or villoglandular)*

This variety was not seen in the Nigerian and only 4 (4·5%) cases were seen in the American negro (Table III). The youngest patient was a 43-year old female and the oldest was also a female of 83 years of age. The average age for both sexes was 58 years.

*Multiple polyposis*

Although 2 cases of benign lymphoid polyposis have been encountered recently in the Nigerian (Williams and Prince, 1975), not a single case of multiple adenomatous polyps has so far been encountered or histologically proven. Ten cases (11·2%) of multiple polyps were found in the American negro. With the exception of the only case of adenomatosis which occurred in a 36-year old female, all the patients were over the age of 50 years. One patient had an infiltrating adenocarcinoma in the midst of the polyps. Histological examination of several polyps revealed varying degrees of admixture of histological types and at various sites (Table IV). The rectum was the commonest site for these multiple polyps. There is no information about familial history in any of the patients or other features of Gardner's syndrome in these patients.

*Juvenile polyps*

This was the commonest type of intestinal polyp in the Nigerian patient, accounting for about 60% of all the polyps encountered (Table II). This was in striking contrast to the frequency of 8 cases out of a total of 89 polyps accounting for about 9% in the American negroes (Table IV). The average ages of the Nigerian and American patients were 13 and 8 years respectively. The youngest Nigerian patient was 3 years, compared with 1 year 5 months in the American negro, while the oldest Nigerian was 41 years, compared with 20 years in the American negro. The majority of this type of polyp occurred in the rectum in both population groups but occasional ones were seen in the sigmoid colon. Multiple juvenile polyposis was not enountered in both population groups.

*Others*

Bilharzial and Peutz Jeghers type of polyp were not encountered in the American negro but an example of each of these was seen in a Nigerian. Two cases of rectal polyps due to carcinoid tumour were seen in American negroes aged 18 and 51 years (Table IV) but this was not encountered in the Nigerian (Table II). One solitary lymphoid polyp and one polyp due to a submucous lipoma, both in the rectum, were seen in the American negro but not in the Nigerian. Lymphoid polyposis has been encountered in 2 Nigerians (Williams and Prince, 1975) and submucous lipoma has been seen in the stomach of Nigerians usually associated with ulceration (Williams, unpublished data). Carcinoid tumour of the rectum and other sites of the intestinal tract has also been encountered in the Nigerian but did not present clinically as polyps. Inflammatory pseudopolyps were not uncommon in the African, including the Nigerian, but this was not included in the present study. Similar inflammatory pseudopolyps were also encountered in 16 American negroes but these were also excluded. The group of non-neoplastic inflammatory polyps in the Nigerian has been described elsewhere (Williams and Prince, 1975).

DISCUSSION

This comparative study reveals the striking differences between the frequencies of the histological types of polyps occurring in both population groups which are ethnologically related. Furthermore, it highlights the relative importance of age in relation to the frequencies of the polyps.

Excluding the group of hamartomatous polyps from the Nigerian group, there were only 3 patients with neoplastic polyps (Table II). This is comparable with the experience of others who have reported similar low frequencies from East and South Africa (Templeton, 1973; Bremner and Ackerman, 1970). The group of neoplastic polyps accounted for 7·5% in the Nigerian compared with about 87·0% in American negroes. It is noteworthy that no instance of neoplastic polyp was encountered in the Nigerian female (Table II). Of possible relevance is the striking disparity between the age adjusted incidence rate in the American negro female for colon cancer which is 23·8/100,000 compared with 0·9, 0·6 and 2·1 in West, East and South African females respectively (Doll *et al.*, 1966, 1970). Although there may be other factors which are significant in the pathogenesis of colon cancer, it would appear that the frequency of neoplastic polyps may be of considerable importance. Evidence is forthcoming from preliminary epidemiological data and information that colonic cancer is relatively low in areas where the frequency of intestinal neoplastic polyps is low (Haenszel and Correa, 1971; Burdette, 1971). The populations with low frequencies of neoplastic polyps and colonic cancer appear to be less sophisticated, including South American Indians, Eskimos, Pygmies of Central Africa, Australian Aborigines and Polynesians of the Pacific. The dietary and faecal composition of these population groups, which are different from those of Caucasians living in the same environment, may also be responsible for this observed relative rarity (Burkitt, 1971).

Another important factor which has not been emphasized enough in the pathogenesis of intestinal polyps is the age structure of the population at risk.

The average ages of all Nigerian males and females with polyps were 26 and 14 years respectively. This is lower than the average age at which any of the neoplastic polyps, which may be antecedent lesions for intestinal carcinomata, usually develop. Of the 81 neoplastic polyps, excluding the 8 juvenile polyps, encountered in the American negro, 59 (72·8%) were in patients over the age of 50 years. This is higher than the average life expectancy of the current African. Of the remaining 22 (27·2%) American negro patients with polyps, there was not a single one under the age of 20 years with an adenomatous or villous type of polyp and there were only 2 patients in the 21–30 year age group. Since the age pyramid structure in the African population reveals a preponderance (>60%) of people living under the age of 30 years, the finding of only 2 polyps (adenomatous (1), villous (1)) and 2 polypoid masses (carcinoid (1), lymphoid (1)) in the American negro under the age of 30 years is perhaps comparable with what was observed in the Nigerian (Williams and Prince, 1975). It is therefore tempting to suggest some possibilities about the epidemiology and pathogenesis of intestinal polyps. The first is that there may be an age specific acquisition pattern with a significant temporal factor for its pathogenesis irrespective of the aetiological agent and the second is that this is perhaps age dependent. This excludes the relatively rare familial cases which are evidently not going to be influenced by age. Of pertinence is the fact that cancer and polyps of the colon were reported to be much less common in American negroes than in Caucasians in recent times (1936–54) (Lawrence, 1936; Quinland and Cuff, 1940; Public Health Monograph, 1956; Steiner, 1954). These racial differences have now almost disappeared (Burkitt, 1971; Alameda Cancer Registry, 1967; Doll *et al.*, 1966; Kovi and Heshmet, 1973). This observed increase may be attributed to several factors, including improved economic standards of the American negro, urbanization with consequent dietary changes, increased life expectancy, changes in the bowel flora and increase of colonic polyps.

The relative rarity of juvenile polyps in the American negro may be a reflection of the age population structure while the abundance of this variety in the Nigerian population may be in support of this suggestion. However, it would appear that this variety is not of significance in the pathogenesis of colon cancer.

Familial polyposis is distinctly rare in Africans (Williams and Edington, 1967; McQuaide and Stewart, 1972; Hutt and Templeton, 1971) and no case was encountered in the American negro or Nigerian in this series. There has been only one reported case of familial polyposis in a South African Bantu (McQuaide and Stewart, 1972) but there are few reports of Gardner's or Peutz Jeghers syndrome in American negroes (Achord and Proctor, 1963; Dodds *et al.*, 1972; Dunning and Ibrahim, 1965; Gordon, Rast and Whelan, 1962). In this study, there were 10 (11·1%) American negroes with multiple polyps. Five patients had multiple (2–3 in number) adenomatous polyps, 3 had admixture of papillary and mixed villoglandular types, 1 had adenomatous and papillary type and there was 1 patient with papillary adenoma of caecum with admixture of papillary and mixed villoglandular polyps of ascending colon. Of the 5 with multiple adenomatous polyps only, there was 1 patient with an infiltrating carcinoma in the vicinity of the polyp. There was no history of familial polyposis in any of these cases and we were not aware of the presence of other features of Gardner's syndrome in any of our cases.

If age is a significant factor, one would expect a rise in the frequency of polyps in the African population groups with increased numbers of people living above the age of 50 years and presenting for medical care. Since intestinal polyps have been shown to be interrelated to colon cancer (Ekelund and Pihl, 1974; Morson, 1971), it is not surprising that the average age for colon cancer in the African is also below 50 years (Williams and Edington, 1967), while in the American negro the peak age frequency of colon cancer is in the seventh decade (Kolade, Chung and White, 1973). Further studies are required, primarily to estimate the frequency or incidence of neoplastic polyps in population groups with low frequency of colorectal cancer and secondarily to find out factors which predispose to its development and its importance in the aetiology and pathogenesis of intestinal cancer. The evidence which can be adduced from this comparative study, though indirect, would suggest that a genetic factor is not significant but age and environmental factors, including diet, may be of importance in the pathogenesis of intestinal neoplastic polyps.

We wish to thank Dr B. Morson and Dr Bussey for their help and advice. This study was supported by grant from the Cancer Research Campaign Fund. We are grateful to Miss K. Wallace for her secretarial help.

### REFERENCES

ACHORD, J. L. & PROCTOR, H. D. (1963) Malignant Degeneration and Metastasis in Peutz Jeghers Syndrome. *Archs intern. Med.*, **111**, 498.

ALAMEDA COUNTY CANCER REGISTRY (1967) *Incidence of Cancer in Alameda County, California*, 1960–64. Berkeley: State of California Department of Public Health.

ARIES, V., CROWTHER, J. S., DRASER, B. S., HILL, M. J. & WILLIAMS, R. E. O. (1969) Bacteria and the Aetiology of Large Bowel Cancer. *Gut*, **10**, 334.

BREMNER, C. G. & ACKERMAN, L. V. (1970) Polyps and Carcinoma of the Large Bowel in the South African Bantu. *Cancer, N.Y.*, **28**, 3.

BURDETTE, W. J. (1971) Identification of Antecedents to Colorectal Cancer. *Cancer, N.Y.*, **28**, 51.

BURKITT, D. P. (1971) Epidemiology of Cancer of the Colon and Rectum. *Cancer, N.Y.*, **28**, 3.

DODDS, W. J., SCHULTE, J. W., HENSLEY, G. T. & HOGAN, W. J. (1972) Peutz Jeghers Syndrome and Gastrointestinal Malignancy. *Am. J. Roentgen.*, **115**, 374.

DOLL, R. (1969) The Geographical Distribution of Cancer. *Br. J. Cancer*, **23**, 1.

DOLL, R., MUIR, C. S., & WATERHOUSE, J. (1970) *Cancer Incidence in Five Countries. A Technical Report*, Vol. II. U.I.C.C. Geneva, Berlin: Springer-Verlag.

DOLL, R., PAYNE, P. & WATERHOUSE, J. (1966) *Cancer Incidence in Five Continents. A Technical Report*. Vol. I. Internat. Un. Cancer. New York, Heidelberg, Berlin: Springer-Verlag.

DUNNING, E. J. & IBRAHIM, D. S. (1965) Gardner's Syndrome. *Ann. Surg.*, **161**, 565.

EKELUND, G. R. & PIHL, B. (1974) Multiple Carcinomas of the Colon and Rectum. *Cancer, N.Y.*, **33**, 1630.

GORDON, W. C., RAST, M. F. & WHELAN, T. J. JR (1962) Gardner's Syndrome. *Ann.Surg.*, **155**, 538.

HAENSZEL, W. & CORREA, P (1971) Cancer of the Colon and Rectum and Adenomatous Polyps. A Review of Epidemiological Findings. *Cancer, N.Y.*, **28**, 14.

HIGGINSON, J. (1967) Etiology of Gastrointestinal Cancer in Man. *Natn. Cancer Inst. Monog.*, **25**, 191.

HILL, M. J., DRASER, B. S., HAWKSWORTH, G., ARIES, V. & WILLIAMS, R. E. O. (1970) Bacteria and Aetiology of Cancer of Large Bowel. *Lancet*, i, 95.

HUTT, M. S. R. & TEMPLETON, A. C. (1971) The Geographical Pathology of Bowel Cancer and Some Related Diseases. *Proc. R. Soc. Med.*, **65**, 962.

KOLADE, S. O., CHUNG, E. B. & WHITE, J. E. (1973) Neoplastic Lesions of the Colon and Ano-rectum in Blacks. *J. Am. med. Ass.*, **65**, 142.

KOVI, J. & HESHMAT, M. Y. (1972) Incidence of Cancer in Negroes in Washington D.C. and Selected African Cities. *Am. J. Epidem.*, **96**, 401.

LAWRENCE, J. C. (1936) Gastrointestinal Polyps. Statistical Study of Malignancy Incidence. *Am. J. Surg.*, **31**, 499.

MCQUAIDE, J. R. & STEWART, A. W. (1972) Familial Polyposis of the Colon in the Bantu. *S. Afr. med. J.*, **46**, 1246.

MORSON, B. C. (1971) Precancerous Conditions of the Large Bowel. *Proc. R. Soc. Med.*, **65**, 959.

PUBLIC HEALTH MONOGRAPH (1956) *Morbidity from Cancer in the United States.*

QUINLAND, W. S. & CUFF, J. R. (1940) Primary Cancer in the Negro. Anatomic Distribution of 300 Cases. *Archs Path.*, **30**, 393.

STEINER, P. E. (1954) *Cancer: Race and Geography.* Baltimore: Williams and Wilkins Co.

TEMPLETON, A. D. (1973) *Tumors in a Tropical Country*. New York, Berlin: Springer-Verlag.

WILLIAMS, A. O. & EDINGTON, G. M. (1967) Malignant Disease of Colon, Rectum and Anal Canal in Western Nigeria. *Dis. Col. Rect.*, **10**, 301.

WILLIAMS, A. O. & PRINCE, D. L. (1975) Intestinal Polyps in the Nigerian African. In the press.

WORLD, HEALTH ORGANIZATION (1975) *Histological Classification of Tumours.* To be published.